# A global dataset of spatiotemporal drought events from reanalysis and hydrological model data for 1980–2024

**DOI:** 10.1038/s41597-026-07750-x

**Published:** 2026-06-29

**Authors:** Vít Šťovíček, Martin Hanel, Rohini Kumar, Vojtĕch Moravec, Yannis Markonis, Carmelo Cammalleri, Jan Řehoř, Miroslav Trnka, Oldrich Rakovec

**Affiliations:** 1 Faculty of Environmental Sciences, Czech University of Life Sciences Prague, Kamýcká 129, Praha, Suchdol 165 00 Czech Republic; 2 Czech Hydrometeorological Institute, Praha, Czech Republic; 3 T. G. Masaryk Water Research Institute, Podbabská 2582/30, 16000, Praha, Czech Republic; 4 Helmholtz Center for Environmental Research (UFZ), Leipzig, Germany; 5 Dipartimento di Ingegneria Civile e Ambientale (DICA), Politecnico di Milano, Milan, Italy; 6 Institute of Geography, Masaryk University, Brno, Czech Republic; 7 Global Change Research Institute of the Czech Academy of Sciences, Brno, Czech Republic; 8 Department of Agrosystems and Bioclimatology, Mendel University in Brno, Brno, Czech Republic

## Abstract

We present a global dataset of spatiotemporally clustered drought events for 1980–2024, derived from daily precipitation, potential evapotranspiration, soil moisture, and surface runoff data. Drought conditions were consistently defined using a 10th percentile threshold and clustered in space and time using a three–dimensional implementation of the Density–Based Spatial Clustering of Applications with Noise (DBSCAN) algorithm. The dataset represents droughts as coherent spatiotemporal events rather than isolated grid–cell anomalies. For each drought event, it provides detailed metadata on spatial extent, temporal duration, severity, and centroid position. By applying a consistent event–detection framework across atmospheric forcing, root–zone soil moisture, and runoff response, the dataset supports systematic analysis of global drought dynamics and compound extremes. The dataset is openly available at https://doi.org/10.5281/zenodo.18292641, providing a reusable resource for climate, hydrology, and hazard research.

## Background & Summary

Droughts are dynamic, multivariate phenomena that evolve across both space and time. Here, we define drought as a dry anomaly relative to local hydroclimatic conditions, rather than an absolute lack of water. This enables comparisons of drought across different climate regions and it motivates the use of locally varying percentile thresholds. Traditional drought indices, typically based on a single hydroclimatic variable such as the Standardized Precipitation Index (SPI), Standardized Precipitation–Evapotranspiration Index (SPEI), or Standardized Runoff Index (SRI), remain widely used to characterize individual drought conditions^1–4^. More recent approaches have introduced multivariate drought indices that integrate additional environmental variables, including the Multivariate Standardized Drought Index (MMSDI), Integrated Drought Index (IDI), and Composite Drought Index (CDI)^5–7^. The need to refine these assessments is highlighted by recent global observations: several studies utilizing gridded drought indices have identified a trend of increasing drought severity and multiyear persistence on a global scale^8–11^.

Despite these global trends, traditional applications of drought indices often characterize droughts as temporally isolated anomalies at individual grid cells or catchments. Such representations are useful for monitoring drought severity, but they do not capture the full spatiotemporal development of drought events (initiation, movement, termination). To address these limitations, recent methodological advances, based on early soil–moisture studies^12,13^, have shifted towards clustering and tracking algorithms that treat droughts as evolving spatiotemporal events^14,15^.

Among these, density–based methods, such as DBSCAN (Density–Based Spatial Clustering of Applications with Noise), group neighboring points into clusters based on local density. This method enables identification of spatially connected drought regions without predefining the number of clusters and filters out noise, defined as data points that do not fall into a dense cluster^16–18^. Extensions such as ST–DBSCAN introduce separate spatial and temporal distance thresholds to support tracking of drought migration and displacement^18–20^. Similarly, 3D DBSCAN treats time as a third dimension alongside latitude and longitude, creating a unified framework for identifying continuous spatiotemporal structures. By jointly considering space and time, 3D DBSCAN provides a comprehensive depiction of drought event evolution, and has been successfully applied to detect extremes using precipitation and temperature anomalies^21–24^.

Other density–based approaches, such as OPTICS (Ordering Points To Identify the Clustering Structure), identify clusters across multiple density levels rather than relying on a single fixed density threshold^18,25^. Instead of producing a single clustering, OPTICS creates an ordered structure of points from which clusters of varying density can be extracted. This approach offers greater flexibility, but increases interpretive complexity. Although recent studies have applied OPTICS to soil moisture anomalies, these applications have been limited to a single variable^26^.

To address this gap, we developed a global dataset of spatiotemporal drought clusters derived from four hydroclimatic variables: soil moisture, runoff, precipitation, and potential evapotranspiration (PET), at 0.5° spatial resolution and daily time step from 1980 to 2024. The selected variables represent different stages of drought propagation through the hydrological cycle^27,28^, from precipitation deficits and evaporative–demand anomalies to soil moisture and runoff deficits.

Collectively, these variables enable analysis of drought propagation and persistence across meteorological, agricultural, and hydrological processes. In contrast to previous studies that primarily focus on single drought metrics, the present framework applies 3D DBSCAN consistently across multiple hydroclimatic variables. This approach enables direct comparison of drought event behavior and propagation throughout the hydrological cycle. Drought conditions for each variable were defined using a locally varying 10th percentile threshold, widely used in both operational drought monitoring and research applications to identify severe drought. It provides a consistent and comparable definition across regions and variables^29–32^.

The resulting dataset serves as a resource for drought attribution studies, hydrological assessments, and machine–learning workflows for hazard monitoring and compound–event analysis^21,33^. By cataloguing events across space and time, the dataset enables global analyses of drought frequency, persistence, and spatiotemporal evolution^21,26,34^. The dataset also supports investigations into links between drought development and large–scale climate variability. Recent advances in artificial intelligence and machine–learning approaches for hydroclimatic extremes prediction increasingly rely on long–term event databases for model training, validation, and benchmarking. In this context, the presented catalogue provides a consistent event–based resource for machine–learning applications in drought detection, prediction, and early warning, while also supporting the development and evaluation of future data–driven forecasting frameworks^19,35–40^.

## Methods

**Fig. 1:**
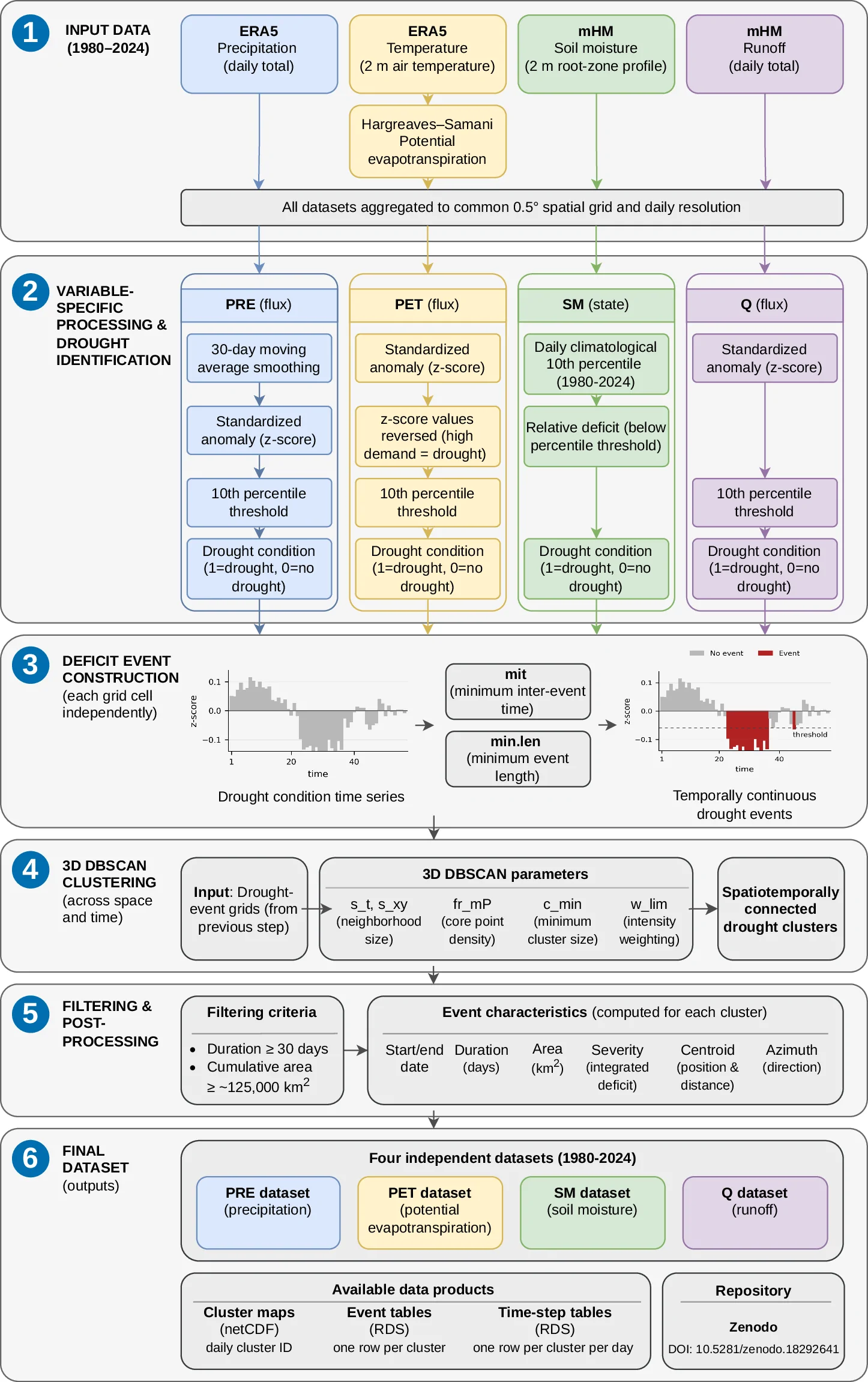
Schematic overview of the drought event dataset generation workflow.

The methodology used to construct a global dataset of spatiotemporal drought events is summarized in Fig. 1. The workflow comprises six consecutive steps: (i) preparation of the hydroclimatic input data, (ii) variable–specific drought identification, (iii) deficit–event construction, (iv) spatiotemporal clustering using 3D DBSCAN, (v) event filtering and characterization, and (vi) generation of the final drought event datasets. The individual steps are described in the following sections.

### Input data

The dataset combines publicly available meteorological reanalysis data with hydrological model outputs generated specifically for this study:

- Daily precipitation and 2–m air temperature were obtained from the ERA5 atmospheric reanalysis^41^ produced by the European Centre for Medium–Range Weather Forecasts (ECMWF) and distributed through the Copernicus Climate Data Store (CDS) at https://cds.climate.copernicus.eu/, openly available under https://doi.org/10.24381/cds.adbb2d47. Specifically, precipitation was obtained from the ERA5 total precipitation variable, and temperature data were from the 2–m air temperature fields available in the ERA5 single–level reanalysis product. ERA5 provides global atmospheric variables at approximately 0.25° spatial resolution and hourly temporal resolution.
- Soil moisture and runoff were obtained from the mesoscale Hydrologic Model (mHM), a distributed, process–based hydrologic model that uses multiscale parameter regionalization to derive effective hydrological parameters at the target spatial scale and simulates key water–cycle components^42,43^. In mHM, the water balance is closed at each grid cell, and all major hydrologic fluxes and state variables are calculated for each cell. Soil moisture represents the average water content over the full 2 m root–zone profile. Runoff was represented by the total runoff component simulated by mHM at the grid–cell scale. The mHM source code is openly available and documented, and the model has been described and evaluated in previous studies^44^. The global mHM setup used here follows the published global–scale mHM modelling framework described by Shrestha *et al*. (2025; Section 3.1)^45^, while the present study generated the specific daily soil–moisture and runoff fields needed for the dataset. As with any large–scale hydrological modelling framework, the resulting soil moisture and runoff estimates are subject to uncertainties arising from meteorological forcing data, model parameterization, structural assumptions, and the representation of regional hydrological processes^42,46^.
- Potential evapotranspiration (PET) was estimated using the Hargreaves–Samani method^47^, calculated from ERA5 temperature variables. The Hargreaves–Samani approach was selected because it requires only temperature inputs, enabling a temporally consistent global PET dataset for the entire study period while avoiding additional uncertainties associated with globally varying radiation, humidity, and wind–speed datasets. The resulting PET product should therefore be interpreted as a consistent representation of atmospheric evaporative–demand anomalies rather than as a definitive estimate of actual evaporation.

Soil moisture, runoff, and PET datasets were restricted to land areas, excluding Antarctica and areas north of 72° N. In contrast, precipitation deficits were analysed globally over both land and oceans. This preserves complete precipitation–deficit events, including cases where clusters originate over oceans and subsequently move onto land, thereby preventing the artificial division of clusters at coastlines. All datasets were aggregated to a common 0.5° grid using bilinear interpolation^48^, ensuring spatial consistency across all hydroclimatic variables. The adoption of a common 0.5° grid enabled direct comparison and overlap analyses among hydroclimatic variables for the 1980–2024 period. However, this spatial resolution primarily captures large–scale drought dynamics and may not adequately represent localized drought variability or small–scale hydrological processes. As a result, the dataset emphasizes persistent and spatially coherent large–scale drought events, rather than localized or short–duration hydroclimatic anomalies.

### Drought event identification

Drought conditions were identified independently for each variable. To suppress short–term variability in individual precipitation events and improve the detection of sustained precipitation deficits relevant to drought development, precipitation was smoothed with a 30–day moving average, a commonly used temporal filtering method in hydroclimatic drought analyses^27^. Soil moisture was treated as a state variable, reflecting longer–term water storage in the root zone, which allows its percentile deficit to be interpreted directly as a measure of agricultural and hydrological drought stress. In contrast, precipitation, PET and runoff were considered fluxes, capturing short–term meteorological forcing and surface response instead^42,43^.

Soil moisture deficits were identified using percentile thresholds computed from long–term daily climatology, with a 30–day smoothing window applied to reduce variability. The 10th percentile of daily soil moisture (1980–2024) was used as a dynamic threshold for each grid cell and calendar day. Daily values below this threshold were expressed as relative deviations:

1 $${\rm{def}}(t)=\frac{SM(t)-S{M}_{p10}(d)}{S{M}_{p10}(d)}\times 100$$

where *SM*(*t*) is the soil moisture value at time step *t* and $S{M}_{p10}(d)$ is the climatological 10th percentile threshold associated with calendar day–of–year *d*, estimated using a moving temporal window around each day of the year. Values below this threshold were classified as drought conditions. The resulting index quantifies the percentage shortfall of root–zone soil water relative to the lower decile of expected conditions. This percentile–based approach aligns with previous mHM–based drought studies^43,49,50^, but is applied here at daily resolution and using day–of–year specific percentiles rather than monthly standardized indices. Using a percentile threshold is consistent with operational drought monitoring practices, where drought severity is defined relative to local climatology (e.g., U.S. Drought Monitor^29^; Intersucho^31^), which ensures interpretability across climate regions. For global–scale applications, percentile thresholds additionally provide a distribution–free framework that avoids uncertainties associated with fitting probability distributions to local hydroclimatic records, which can be particularly problematic in arid and hyperarid regions^51–53^.

For precipitation, potential evapotranspiration, and runoff, anomalies were standardized to z–scores over the 1980–2024 baseline to ensure comparability across regions and variables. Z–scores were calculated by subtracting the long–term mean from each value and dividing by the standard deviation. For PET, the sign of the z–scores was reversed prior to threshold calculation so that higher evaporative demand was represented as drought conditions. Drought conditions were then defined as days where the standardized anomaly fell below the grid–cell specific 10th percentile of the z–score distribution. Unlike SPI^1,2^ or SPEI^3^, which require fitting probability distributions to estimate standardized drought indices, the z–score approach provides a simpler anomaly–based representation that does not require fitting a parametric probability distribution^29,54^. Z–score remain widely used in global drought assessments and comparative analyses across climate regimes^29,54,55^.

Within this drought identification framework, the climatological 10th percentile serves as the consistent threshold for drought identification across all variables. Relative deficits and standardized anomalies (z–scores) were then used to quantify the magnitude of drought conditions relative to long–term hydroclimatic variability, rather than to define drought occurrence. Employing a common percentile threshold enabled consistent drought identification across variables with differing units and temporal variability.

Next, a custom “deficit volume calculation function” was applied to flag all days where the standardized anomaly fell below the 10th percentile drought threshold and to group consecutive deficit days into continuous events. Short interruptions and brief events were adjusted according to the control parameters described as follows:

- **mit** (*minimum inter–event time*): the allowable number of consecutive non–deficit days that may occur within a larger deficit period without breaking the event. This parameter accounts for brief fluctuations, such as isolated rainfall, that do not interrupt drought persistence.
- **min.len** (*minimum length*): the minimum duration required for a continuous (corrected) deficit period to be classified as a drought event. This criterion ensures retention of only sustained dry periods.

**Fig. 2:**
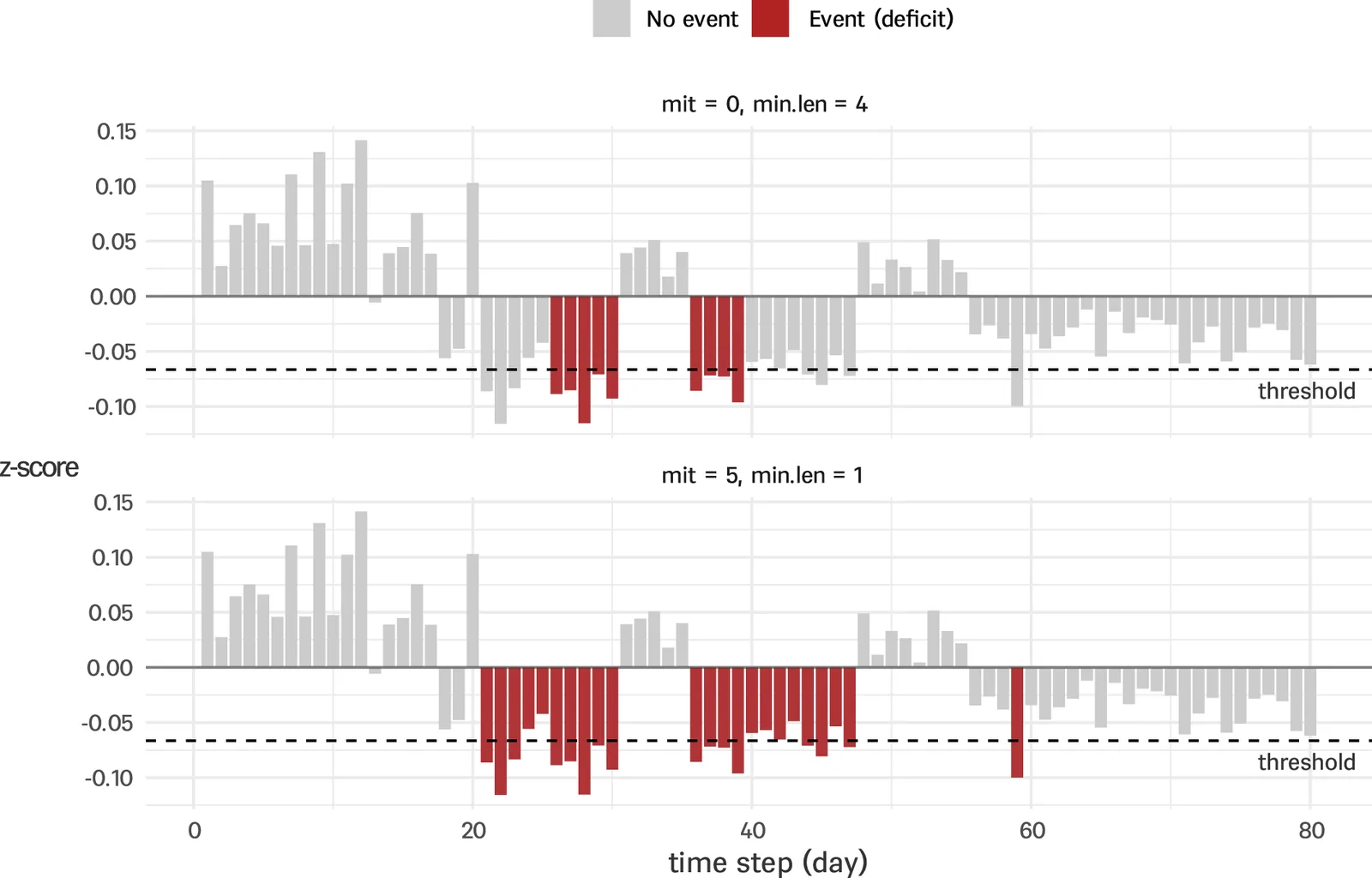
Effect of *mit* and *min.len* parameters on detected drought events using an example runoff time series. Bars represent standardized anomalies (z–scores) over time. Red columns denote periods classified as drought events according to the applied *mit* (minimum inter–event time), *min.len* (minimum event length) settings and selected threshold, while grey columns indicate non–event periods.

Figure 2 demonstrates the influence of *mit* and *min.len* on drought event detection using an example runoff time series. In the top panel (mit = 0, min.len = 4), short interruptions divide the sequence of deficits, resulting in the classification of only longer continuous periods as drought events. Conversely, the bottom panel (mit = 5, min.len = 1) permits brief non–deficit days and shorter durations, leading to identification of longer and more frequent drought events. Each drought event was characterized by its duration and cumulative deficit (intensity), enabling spatiotemporal tracking in subsequent clustering. Threshold and anomaly computations were primarily conducted using Climate Data Operators (CDO)^48^, which efficiently processed large NetCDF files (tens of GB in this project). All further analyses, including 3D DBSCAN clustering, were performed in R^56^.

### Spatiotemporal clustering via 3D DBSCAN

Drought events were identified as connected regions in space and time using a 3D DBSCAN algorithm^14,16,17^. Each input pixel was characterized by its spatial coordinates, temporal index, and severity weight, derived from the standardized deficit magnitude at that grid cell and time step. Clustering was performed in two steps: (i) a spatial DBSCAN applied to each time step, followed by (ii) a full 3D DBSCAN across space and time.

The DBSCAN parametrization followed the methodology of Cammalleri *et al*.^17^ with settings selected to balance event detection and noise suppression (Table 1). The key parameters were as follows:

- **Neighborhood size**: The smallest meaningful spatial (*s_xy* = 3) and temporal (*s_t* = 3) windows were used to limit the risk of merging adjacent but unrelated events and to enable detection of small, localized droughts.
- **Core point density**: A fractional minimum number of points (*fr_mP* = 0.25) within the search window was required to ensure compact and well–defined clusters.
- **Minimum cluster size**: A filter of *c_min* = 50 pixels (≈125 000 km^2^ at the equator with 0.5° spatial resolution) was applied to exclude spurious or fragmented regions.
- **Intensity weighting**: The parameter *w_lim* (with *w_exp* = 1.5) was varied to adjust the influence of drought severity. This adjustment enabled prioritization of impactful events when the number of clusters exceeded expected cataloguing capacity.

**Table 1:** 3D DBSCAN Parameters Description.

Parameter	Description
s_xy	Spatial neighborhood size in grid cells, forming a window centered on each pixel.
eps_xy	Spatial distance threshold used in DBSCAN, computed as the Euclidean distance across the s_xy kernel. Controls spatial proximity.
s_t	Size of the temporal window (in days) for 3D clustering.
eps_t	Temporal distance threshold used in 3D clustering, set equal to eps_xy for consistent scaling across space and time.
fr_mP	Fraction used to compute the minimum number of points (minPts) required to form a dense region. Applied to the number of potential neighbors in the search window in both 2D and 3D steps.
mP_xy	Minimum number of spatial neighbors in 2D required to form a cluster. Derived from fr_mP and s_xy.
mP_t	Minimum number of points in 3D (space–time) required to form a cluster. Derived from fr_mP, s_xy, and s_t.
c_min	Minimum number of pixels for retaining 2D clusters before performing full 3D clustering. Helps filter out small or fragmented regions.
w_lim	Controls the intensity threshold for weighting drought magnitude.
w_exp	Controls the steepness of the weight response to drought intensity.
www	Weight assigned to each pixel using the defined weight function. Derived from input deficit value and conversely from w_lim and w_exp.

**Fig. 3:**
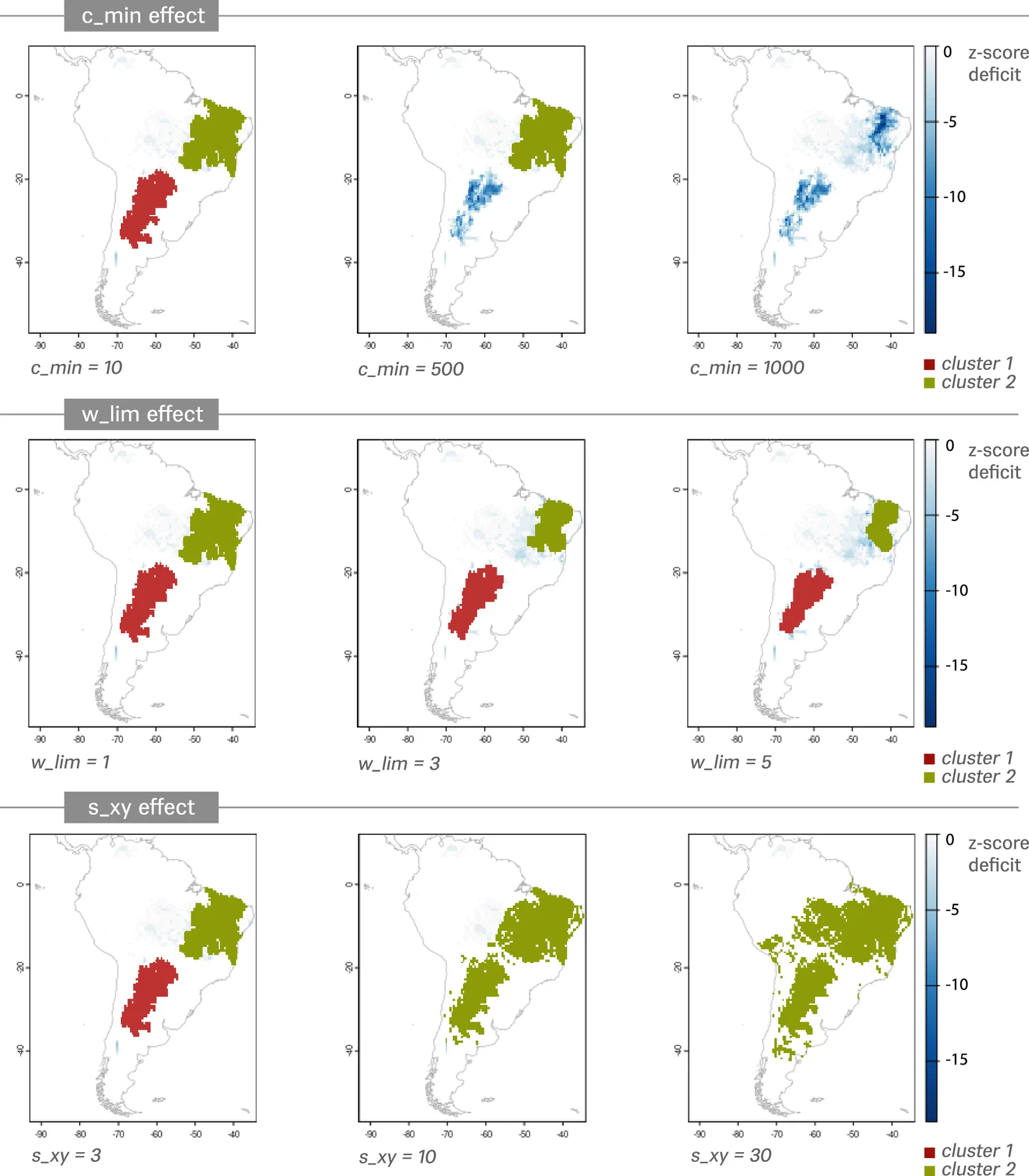
The effect of varying the minimum cluster size (*c_min*), intensity threshold for weighting drought magnitude (*w_lim*), and spatial neighborhood radius (*s_xy*) on drought event detection. Colored areas show clusters identified by 3D DBSCAN, overlaid on z–score deficits from 13.03.2011 over South America. Parameter settings strongly influence the size, extent, and continuity of detected clusters.

The impact of these parameters on event detection is demonstrated in Fig. 3, which illustrates how 3D DBSCAN identifies clusters from soil moisture deficits over South America on March 3, 2011. Increasing c_min eliminates smaller clusters, while increasing w_lim assigns greater weight to pixels with larger z–score deficits, increasing their likelihood of inclusion in clusters. A higher s_xy value expands the search radius, permitting more neighboring deficit pixels to be grouped within the same cluster. All clustering analyses were conducted using the dbscan() function from the R package *dbscan*^57^. To efficiently process large datasets, clustering was parallelized in R using the *parallel*^56^, *doSNOW*^58^, and *foreach*^59^ packages. Each thread accessed NetCDF input files independently via the *ncdf4* package^60^. After temporal stacking of pixel–level drought flags, 3D DBSCAN clustering was applied to identify individual drought events, assigning each pixel to a single cluster ID.

### Postprocessing

A postprocessing workflow then summarized the pixel–level cluster information into event–level characteristics, including the spatial extent, duration, severity, and temporal evolution of each drought event. To focus on persistent events, we retained only clusters lasting at least 30 days (10 days for potential evapotranspiration, for which a longer minimum–duration criterion would exclude most detected events; see Supplementary Note 1, Table S1). For each cluster, the following attributes were derived:

- **Event–level attributes**: start and end dates, total duration, pixel count, and median spatial/temporal centroids.
- **Evolution attributes**: daily centroid position, daily cluster area (km^2^), cumulative severity, and minimum/maximum extent.
- **Movement attributes**: centroid displacement calculated with the Vincenty’s formulae, azimuth of movement (using rhumb line), and cardinal direction of movement (N, NE, E, etc.).

The resulting clusters and derived attributes were exported as global NetCDF files and corresponding R catalogue tables for each variable, as described in the following section.

## Data Records

The complete drought–event dataset is publicly available on Zenodo^61^ at https://doi.org/10.5281/zenodo.18292641. The peer–reviewed version described in this paper corresponds to version 1.0.2 of the repository. It consists of two complementary components for each of the four analyzed variables—precipitation (PRE), potential evapotranspiration (PET), runoff (Q) and soil moisture (SM):

- **NetCDF files**: Four global gridded files that store the 3D DBSCAN cluster identification for each variable at 0.5° spatial and daily temporal resolution (1980–2024) named:
  - PRE_ERA5_3Ddbscan_global_res0p5_1980_2024_1day.nc
  - PET_ERA5_3Ddbscan_global_res0p5_1980_2024_1day.nc
  - Q_mHM_3Ddbscan_global_res0p5_1980_2024_1day.nc
  - SM_mHM_3Ddbscan_global_res0p5_1980_2024_1day.nc
  Each file provides, for every grid cell and day, the integer cluster identifier corresponding to the drought event detected at that location and time.
- **R catalogue tables**: Two accompanying R data files (.rds) per variable that summarize cluster characteristics in tabular form:
  - summary_ < var > .rds—one record per drought event, describing overall event-level properties.
  - stats_ < var > .rds—daily time-step records for each event.

The parameters contained in these files are detailed in Table 2.

**Table 2:** Description of the variables contained in the stats_<var>.rds and summary_<var>.rds files.

summary_<var>.rds	
Parameter	Description
cluster_id	Unique identifier of the drought event (matches stats_<var>.rds).
start_index	First time step of the cluster (integer index).
end_index	Last time step of the cluster (integer index).
duration_days	Total cluster duration in days (or time steps).
pixel_count	Total number of pixels that belong to the cluster across its full duration.
lat_cen	Median latitude of the cluster centroid (degrees).
lon_cen	Median longitude of the cluster centroid (degrees).
severity_total	Sum of drought intensity (weighted deficit volume) over the entire cluster lifetime.
area_max_km2	Maximum spatial area of the cluster (km^2^) across all time steps.
area_min_km2	Minimum spatial area of the cluster (km^2^) across all time steps.
**stats_.rds**	
**Parameter**	**Description**
cluster_id	Unique cluster identifier (integer).
time_index	Time step index (integer).
lon_median	Median longitude of all cluster pixels (degrees).
lat_median	Median latitude of all cluster pixels (degrees).
area_km2	Area of the cluster at this time step (square kilometers).
severity	Sum of drought intensity (weighted deficit volume) across all pixels at this time step.
dist_km	Distance traveled by the cluster centroid since the previous time step (kilometers).
azimuth_deg	Bearing of centroid displacement since previous time step (degrees, 0° = north, clockwise).
direction	Cardinal direction of centroid movement (e.g., N, NE, E,…).

**Fig. 4:**
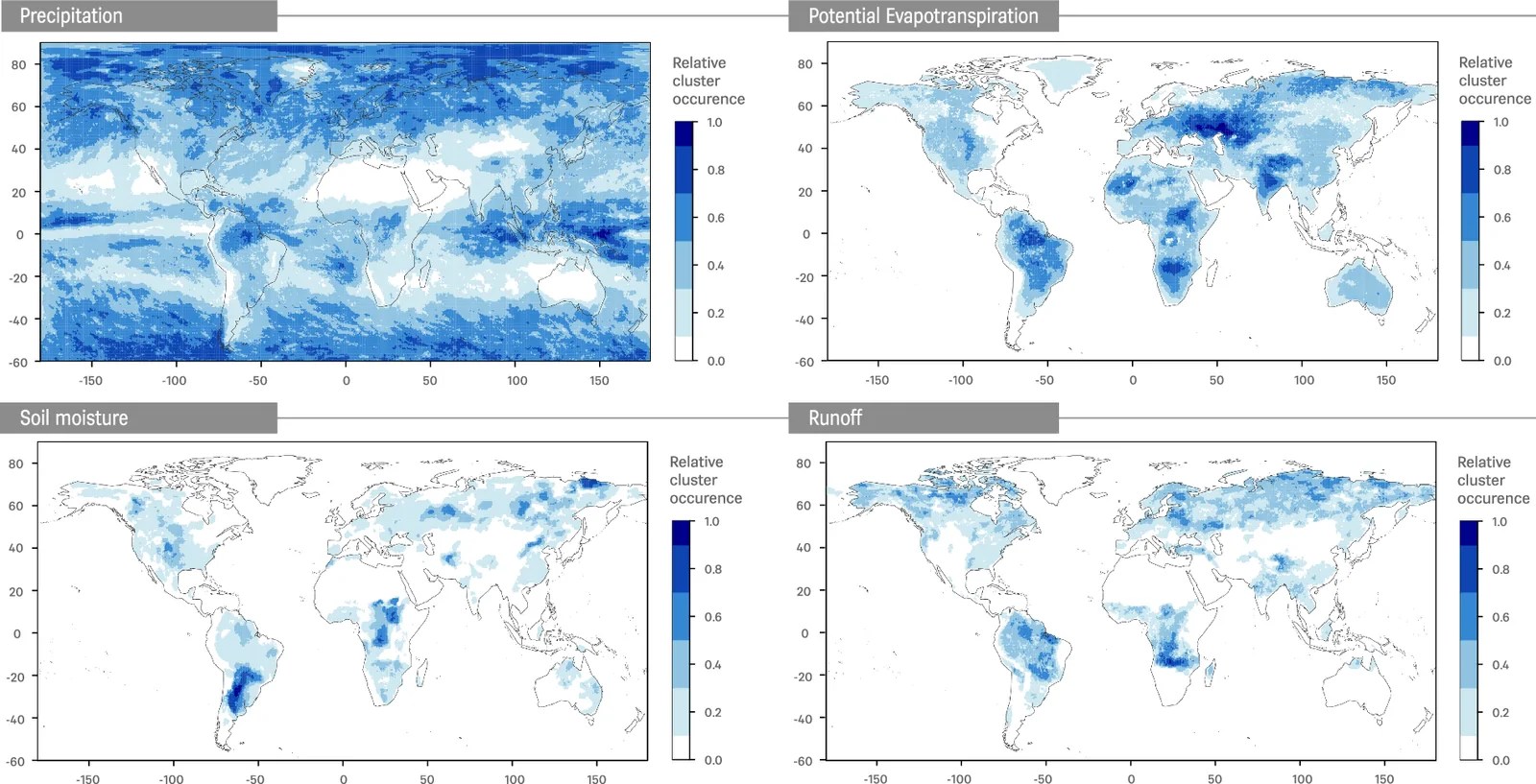
Spatial distribution of relative cluster occurrence for each variable (1980–2024). Maps show the relative frequency with which each grid cell was part of a spatiotemporal drought cluster, normalized to the maximum hotspot for each variable (0 = no cluster occurrence, 1 = highest occurrence). Results are presented for runoff, soil moisture, potential evapotranspiration, and precipitation over the period 1980–2024. Darker colors highlight regions that repeatedly contributed to drought clusters relative to other areas. Precipitation clusters include both land and ocean grid cells, whereas soil moisture, runoff, and PET clusters are shown only over land.

Together, the NetCDF maps and the R catalogue tables form a global database of drought events. This database provides the basis for analysing drought evolution, magnitude, severity, and spatial dynamics across different variables and time scales. In total, the dataset includes 4315 precipitation (of which 1123 occurred exclusively over the ocean), 1724 potential evapotranspiration, 813 runoff and 715 soil moisture clusters. These differences reflect the distinct response times of the variables, where precipitation and PET exhibit high short–term variability, while soil moisture and runoff integrate conditions over longer periods, resulting in fewer but generally more persistent events. Heatmaps of cluster occurrence are shown in Fig. 4, illustrating the spatial patterns and frequency of identified drought events across variables. Precipitation clusters, unlike the other variables, were also derived over oceans, with pronounced hotspots in the tropics and high latitudes. Potential evapotranspiration clusters show strong activity over eastern Europe and central Asia, as well as notable hotspots in South America and southern Africa. Soil moisture clusters are more spatially confined, with concentrations primarily in South America and central Africa. Runoff clusters exhibit a broader distribution, occurring relatively evenly across the higher latitudes of the Northern Hemisphere, as well as prominently in South America and central Africa.

## Technical Validation

Validation of global spatiotemporal drought datasets is inherently challenging due to the absence of globally consistent benchmark observations and universally accepted evaluation metrics for event–based hydroclimatic clustering^62^. Therefore, validation of the presented dataset focused on three complementary aspects: (i) robustness of drought–deficit identification, (ii) sensitivity of the clustering methodology to parameter settings, and (iii) consistency with existing drought datasets and previously documented drought–event behaviour.

### Sensitivity of deficit condition parameters *(mit, min_len)*

We evaluated how the identification of drought conditions responds to the configuration of the deficit–volume algorithm, particularly the minimum inter–event time (*mit*) and minimum event length (*min.len*) parameters. These parameters directly influence the temporal continuity of deficit conditions used as input for the clustering algorithm by allowing short interruptions within drought periods and filtering short–lived anomalies unlikely to represent persistent drought processes. Figure 2 illustrates on the example of runoff time series how varying these parameters affects deficit delineation and thus the resulting 3D DBSCAN clustering behaviour, including event fragmentation, persistence, and cluster continuity. Sensitivity analyses presented in the Supplementary Material further demonstrate how the interaction between *mit*, *min.len*, temporal resolution, and variable–specific behaviour influences drought–event representation. Based on the sensitivity statistics summarized in Table S1 and the representative event examples shown in Figures S1–S4, the selected parameter configuration preserves persistent drought periods while minimizing artificial fragmentation and over–aggregation of clusters.

### Sensitivity to percentile–based drought–deficit thresholds

Next, additional sensitivity analyses were performed to evaluate the influence of percentile–based drought–deficit thresholds on the resulting spatiotemporal drought clusters (Supplementary Note 2, Figures S5–S7 and Table S2). Increasing the percentile threshold increases the number of grid cells classified as drought–affected and therefore increases the spatial and temporal connectivity of deficit conditions supplied to the 3D DBSCAN algorithm. As illustrated in Figure S5, higher thresholds (e.g., the 20th percentile) produced substantially broader and more spatially continuous drought regions, whereas stricter thresholds progressively reduced drought extent while preserving the dominant large–scale hotspot patterns of recurrent drought. Although the median duration and median area of detected events remained relatively stable across tested thresholds (Table S2), substantial differences emerged in the persistence structure of the resulting drought catalogues. In particular, the 20th percentile threshold produced a substantially larger number of multi–year drought events and several extremely persistent clusters exceeding 1000 days in duration (Figure S6). These persistent clusters likely resulted from excessive temporal and spatial connectivity of deficit conditions. As a result, physically distinct drought episodes merged into single spatiotemporal events. Conversely, very strict thresholds (1st––2nd percentiles) substantially reduced the total number of detected events and increased fragmentation of large–scale drought structures (Figures S6, S7). Comparisons across thresholds (Figure S7) showed that the main drought cores remained spatially consistent. Differences primarily occurred along the temporal and spatial margins of events. In several representative cases, the 10th percentile threshold preserved the major drought structures identified under the 20th percentile definition while separating very long continuous clusters into shorter drought events. Based on these analyses, the 10th percentile threshold was selected for the final dataset as a compromise between preserving persistent large–scale drought structures and minimizing excessive cluster merging and unrealistically long event persistence. Consistent with the objectives of the dataset, the selected configuration emphasizes persistent and spatially coherent large–scale drought events rather than localized or short–lived hydroclimatic anomalies.

### Sensitivity of 3D DBSCAN clustering framework to parameter configuration

We additionally assessed the sensitivity of the 3D DBSCAN clustering framework to different parameter configurations. Since density–based clustering algorithms are inherently sensitive to parameter selection, experiments were performed using multiple values of the spatial neighbourhood size (*s_xy*) and the intensity–weighting threshold (*w_lim*). Both daily and 10–day temporal resolutions were tested to evaluate the stability of the resulting drought clusters across different temporal aggregations. Although the total number and size of detected events varied between parameter combinations, the dominant large–scale spatiotemporal drought patterns remained generally consistent, indicating robust event detection across parameterizations. Representative examples of clustering sensitivity are shown in Fig. 3.

### Comparison of clustering outputs across input datasets

Finally, the resulting drought clusters were compared with independent drought datasets and catalogues. In particular, soil moisture drought clusters were evaluated against the global catalogue of Řehoř *et al*.^26^, which applied the OPTICS algorithm to the same underlying soil moisture data. Despite methodological differences, both datasets show similar spatial hotspot distributions and comparable timing of major multi–year drought periods, suggesting that the identified events represent robust large–scale drought dynamics rather than methodological artefacts. Additional validation was performed by comparing the dataset against several well–documented historical drought events reported in the literature. The dataset successfully captures major drought episodes including the 1988 North American drought^63^, the 2003 European drought and heatwave^64^, the 2010 Russian drought^65^, the 2011–2017 California drought^66^, the 2012 United States Midwest drought^67^, the 2015–2016 Southern African drought^68^, the 2018–2020 Central European drought^50^, the 2010–2011 drought in China^69^ or 2023–2024 Amazon drought^70^. The successful identification of these geographically diverse and well–documented drought episodes provides further confidence that the dataset captures physically meaningful large–scale drought dynamics, which is in line with the objective of the dataset to reproduce exact drought boundaries at local scales, but rather to provide a globally consistent representation of the timing, persistence, extent, and evolution of major drought events across multiple hydroclimatic variables.

Additional comparisons using alternative root–zone soil moisture datasets GLEAM v4.2^71^ and SoilClim^72–74^ are provided in Supplementary Note 3 (Figure S8 and Table S3). GLEAM is an observationally constrained root–zone soil moisture product with vegetation–dependent rooting depths, whereas SoilClim is a process–based soil–water balance model representing soil moisture within a fixed 2 m soil profile. All datasets cover the 1980–2024 period and were processed using the same z–score–based drought definition and identical clustering parameters, ensuring methodological consistency and direct comparability among drought–event catalogues. Despite differences in the underlying soil moisture products, all three datasets show similar hotspot distributions and timing of major drought episodes. Furthermore, the resulting catalogues exhibit remarkably consistent median event durations (55–59 days) and comparable median event areas, suggesting that the identified large–scale drought patterns are robust across different soil moisture datasets. Because the mHM soil moisture variable represents the average water content over the full 2 m root–zone profile, these comparisons should be interpreted as large–scale consistency checks of drought patterns and event timing rather than as direct validation of the full mHM soil moisture profile.

## Supplementary information

Supplementary Information

## Data Availability

All drought-event datasets generated in this study are openly available through Zenodo^61^ at https://doi.org/10.5281/zenodo.18292641. The peer-reviewed version associated with this manuscript is version 1.0.2. The repository contains the full set of global 3D DBSCAN drought cluster maps (NetCDF) and the accompanying R catalogue tables for all four hydroclimatic variables. Future updates may be released as new repository versions, to extend the database, ensuring the dataset remains a living resource for climate, hydrology, and risk-assessment.

## Code availability

For this study, a custom 3D DBSCAN script was developed to identify and track spatiotemporal drought events at the global scale. The implementation builds on the original three–dimensional DBSCAN framework proposed by Cammalleri & Toreti^17^, with modifications and extensions tailored to multivariate hydrological data and large–scale spatiotemporal analysis. The peer-reviewed version of script, named *3DDBSCAN_v1.0.R*, is included in the associated Zenodo dataset repository^61^ and is publicly available at https://doi.org/10.5281/zenodo.18292641.
