# Supplementary material for: A global dataset of spatiotemporal drought events from reanalysis and hydrological model data for 1980–2024
Source: Sci Data. 2026 Jun 29;13:1319. doi: 10.1038/s41597-026-07750-x (PMC13582919; doi:10.1038/s41597-026-07750-x)
Supplement: Supplementary file 1 [file 41597_2026_7750_MOESM1_ESM.pdf]

Supplementary Information

Contents

Supplementary Note 1 Sensitivity of deficit condition parameters *(mit, min_ len)* **2**
1.1 Effect of varying *mit* . . . 2
1.2 Effect of varying *min_ len* . . . 3
1.3 Combined effect of *mit* and *min_ len* . . . 4
1.4 Reference cluster evolution from daily data . . . 5
1.5 Summary statistics across parameter combinations . . . 6

Supplementary Note 2. Sensitivity to percentile-based drought-deficit thresholds **7**
2.1 Effect of varying deficit . . . 7
2.2 Summary statistics across deficit definitions . . . 8
2.3 Effect of varying deficit thresholds on drought-event persistence . . . 8
2.4 Cluster persistence across deficit thresholds . . . 9

Supplementary Note 3. Comparison of clustering outputs across input datasets **10**
3.1 Sensitivity of cluster occurrence to input datasets . . . 11
3.2 Summary statistics across different datasets . . . 12

Supplementary Note 1 Sensitivity of deficit condition parameters *(mit, min_len)*

Deficit conditions were constructed using two parameters: (i) minimum event length (min_len), defining the minimum number of consecutive deficit time steps required for an event, and (ii) maximum interruption time (mit), defining the number of consecutive non-deficit time steps allowed within an event.

A sensitivity analysis was performed using potential evapotranspiration (PET) deficits derived from ERA5, aggregated to 10-day intervals. The analysis evaluates how parameter choices influence (i) the spatial distribution of deficit pixels and (ii) the resulting 3D DBSCAN clusters.

Figures S1–S3 illustrate the effect of varying mit and min_len independently and jointly for a representative timestep (June 2022). Figure S4 provides a reference based on daily data from the final catalogue, showing the temporal evolution of clusters without temporal aggregation. Table S1 summarizes the impact of parameter combinations on cluster statistics over the full 1980–2024 period.

1.1 Effect of varying *mit* (minimum inter-event time)

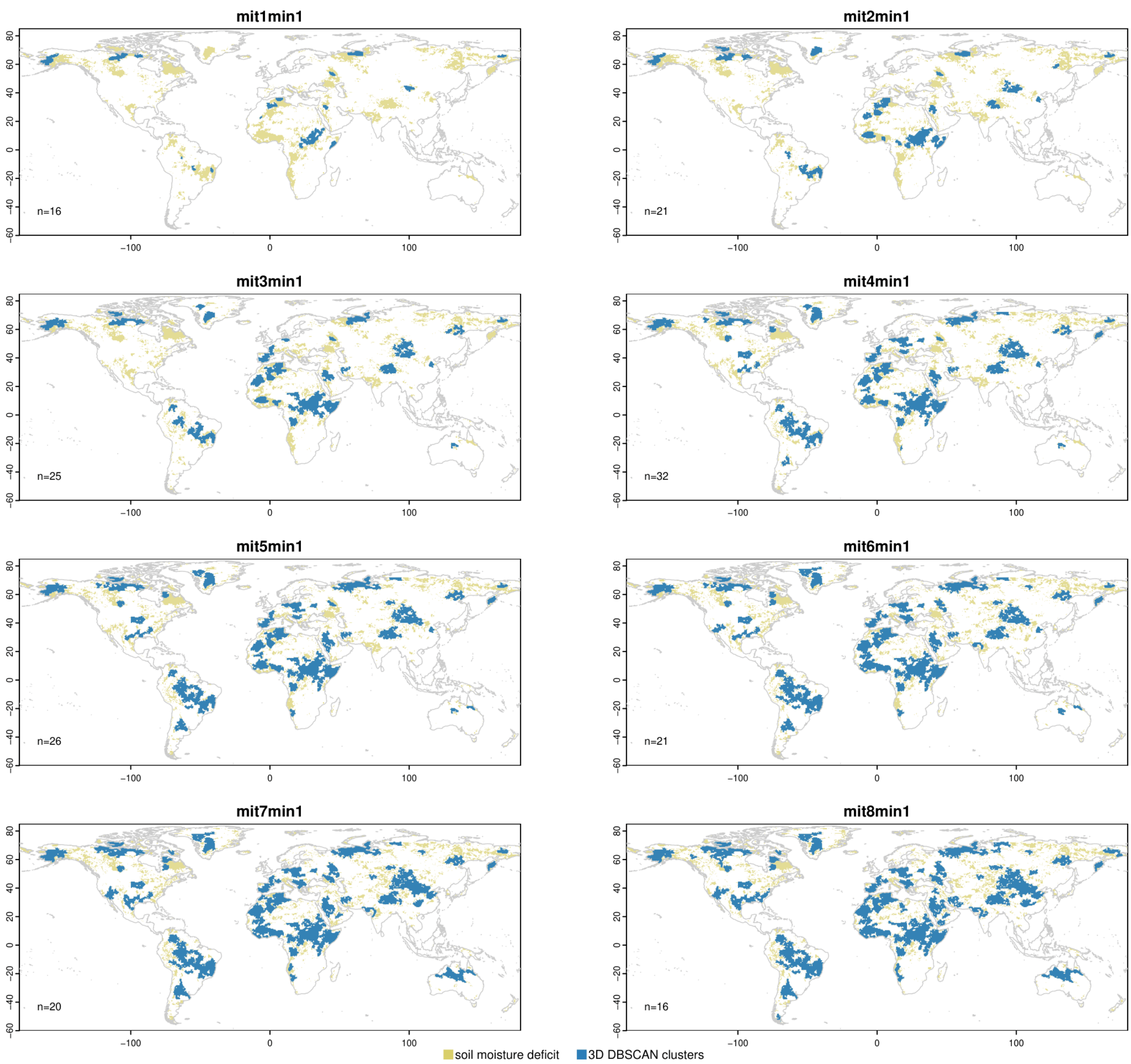

Figure S1: Spatial distribution of deficit pixels (yellow) and resulting 3D DBSCAN clusters (blue) for increasing values of *mit* (1–8), while *min_len* is fixed at 1. The number of detected clusters (n) is indicated in each panel. Increasing *mit* allows short interruptions in the deficit signal to be included within events, leading to changes in cluster extent and connectivity.

1.2 Effect of varying *min_ len* (minimum length)

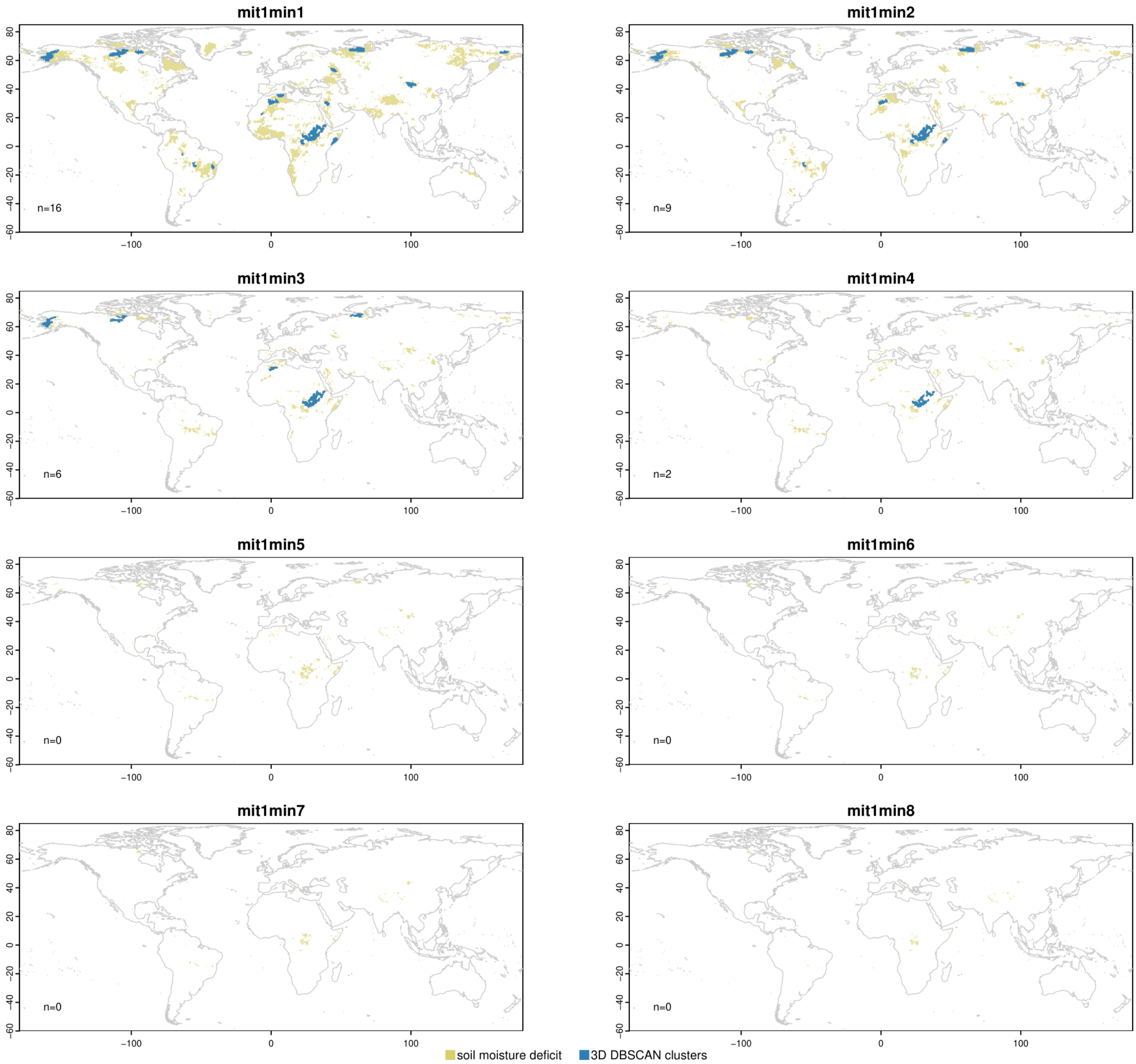

Figure S2: Spatial distribution of deficit pixels (yellow) and resulting 3D DBSCAN clusters (blue) for increasing values of *min_ len* (1–8), while *mit* is fixed at 1. The number of detected clusters (n) is indicated in each panel. Increasing *min_ len* restricts deficit conditions to temporally persistent signals, reducing the number of retained deficit pixels and associated clusters.

1.3 Combined effect of *mit* (minimum inter-event time) and *min_len* (minimum length)

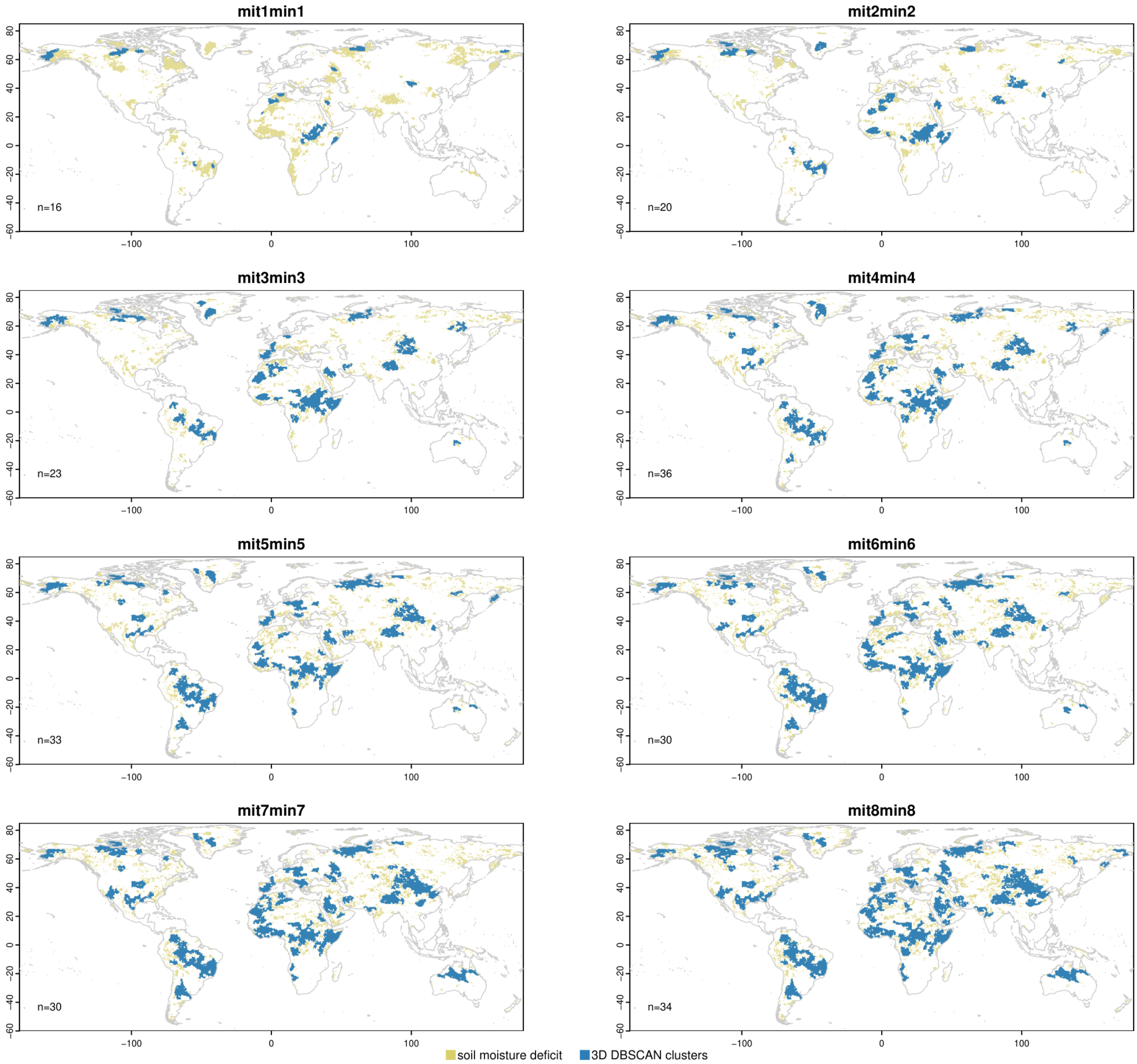

Figure S3: Spatial distribution of deficit pixels (yellow) and resulting 3D DBSCAN clusters (blue) for simultaneously increasing *mit* and *min_len* (1–8). This setup illustrates the combined influence of allowing interruptions (*mit*) while enforcing persistence (*min_len*) on the resulting cluster patterns and counts.

1.4 Reference cluster evolution from daily data

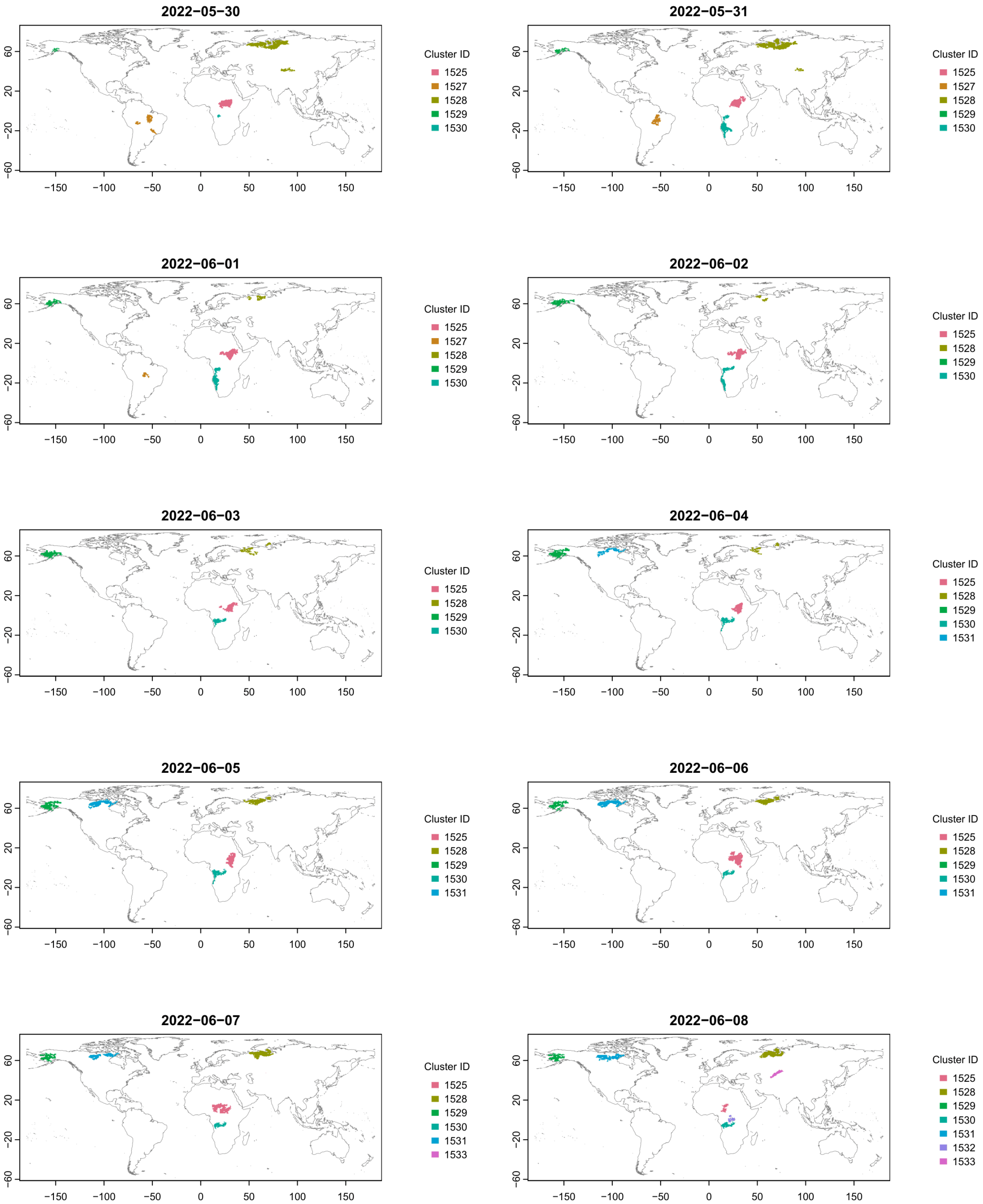

Figure S4: Temporal evolution of PET-based drought clusters derived from daily data in the final catalogue for the period 30 May 2022 to 8 June 2022. Cluster IDs are shown for each timestep. This reference illustrates the continuous movement and evolution of clusters without temporal aggregation as in examples in Figures S1-S3.

1.5 Summary statistics across parameter combinations

Table S1: Summary of clustering results for different combinations of *mit* and *min_len* over the full 1980–2024 period. The table reports the total number of clusters, average cluster duration. The final two rows provide reference values from the final catalogue in its current form with filtering events that are at least 10 days long and from eventual filtering events being long at least 30 days.

mit	min_len	number of clusters	average cluster duration
1	1	2738	39.1
1	2	1782	39.2
1	3	1320	38.6
1	4	510	46.7
1	5	195	55.3
1	6	62	67.6
1	7	32	78.4
1	8	16	90.6
2	1	3887	46.2
3	1	4287	57.4
4	1	4303	70.5
5	1	4107	83.3
6	1	3844	95.7
7	1	3513	109.6
8	1	3199	124.2
2	2	3422	46.4
3	3	3845	58.3
4	4	3524	78.2
5	5	3206	99.5
6	6	2930	120.5
7	7	2679	144.4
8	8	2530	165.6
Current catalogue version *mit = 1, min_len = 1 (daily time step)*			
clusters $\geq$ 10 days		1724	16.3
clusters $\geq$ 30 days		69	44.4

Supplementary Note 2. Sensitivity to percentile-based drought-deficit thresholds

Sensitivity analyses were performed using multiple percentile-based drought-deficit thresholds (20th, 10th, 5th, 2nd, and 1st percentiles) to evaluate the influence of deficit definition on the resulting spatiotemporal drought clusters. Increasing the percentile threshold increases the fraction of grid cells classified as drought-affected and therefore increases the spatial and temporal connectivity of deficit conditions supplied to the 3D DBSCAN clustering framework.

Figure S5 shows the relative frequency of drought-cluster occurrence for each threshold over the 1980–2024 period. Although stricter thresholds progressively reduce the spatial extent and persistence of detected drought conditions, the dominant large-scale hotspot patterns remain generally consistent across all tested definitions. Table S2 summarizes the resulting catalogue characteristics, including the number of detected clusters, median duration, median event area, event severity, and the occurrence of persistent multi-year droughts.

Figure S6 compares the duration structure of the resulting catalogues and demonstrates substantial differences in event persistence among thresholds, particularly for the longest drought events. Figure S7 further illustrates how decreasing the deficit threshold progressively subdivides large persistent clusters into smaller and less temporally continuous drought structures while generally preserving the dominant drought cores.

2.1 Effect of varying deficit

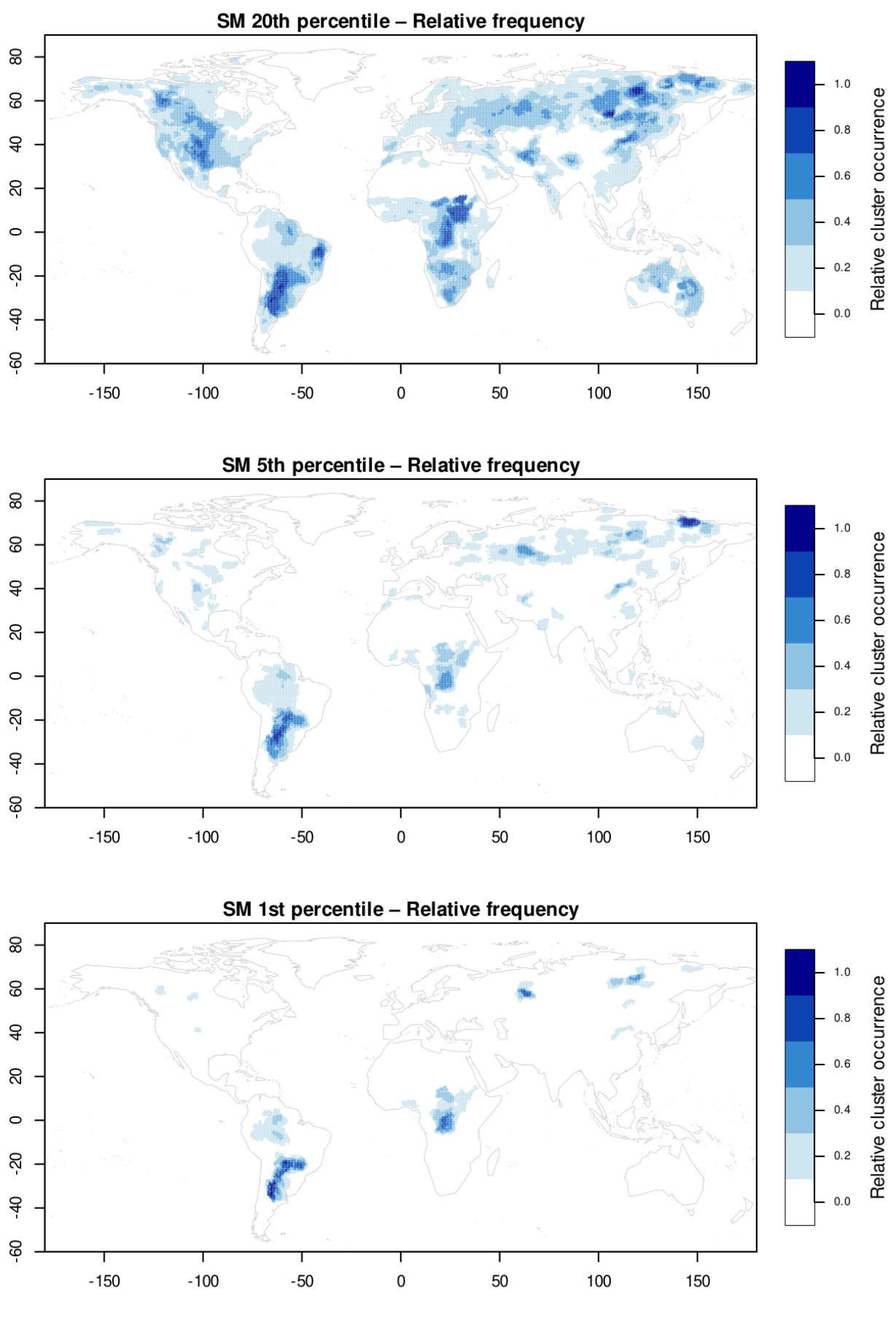

Figure S5: Relative frequency of soil moisture drought-cluster occurrence derived using different percentile-based deficit thresholds (20th, 10th, 5th, 2nd, and 1st percentiles) for the 1980–2024 period. Maps show the fraction of time steps during which individual grid cells were part of drought clusters lasting at least 30 days. The comparison illustrates how stricter deficit thresholds progressively reduce the spatial extent and persistence of detected drought events while preserving the dominant large-scale drought hotspot patterns.

52 2.2 Summary statistics across deficit definitions

Table S2: Summary characteristics of drought-event catalogues derived using different deficit thresholds. Lower deficit thresholds generally resulted in fewer but more persistent drought events, whereas higher thresholds produced a larger number of shorter-duration events.

Deficit	Number of clusters	Median duration (days)	Median area (km^2)	Mean severity	Events > 1 year	Events < 3 months
def20	1328	68.0	415823	166653856	61	811
def10	715	63.0	383146	64654881	23	456
def5	307	63.0	347588	29644180	7	194
def2	60	68.5	345398	9226784	3	38
def1	46	75.5	421083	4750250	3	28

53 2.3 Effect of varying deficit thresholds on drought-event persistence

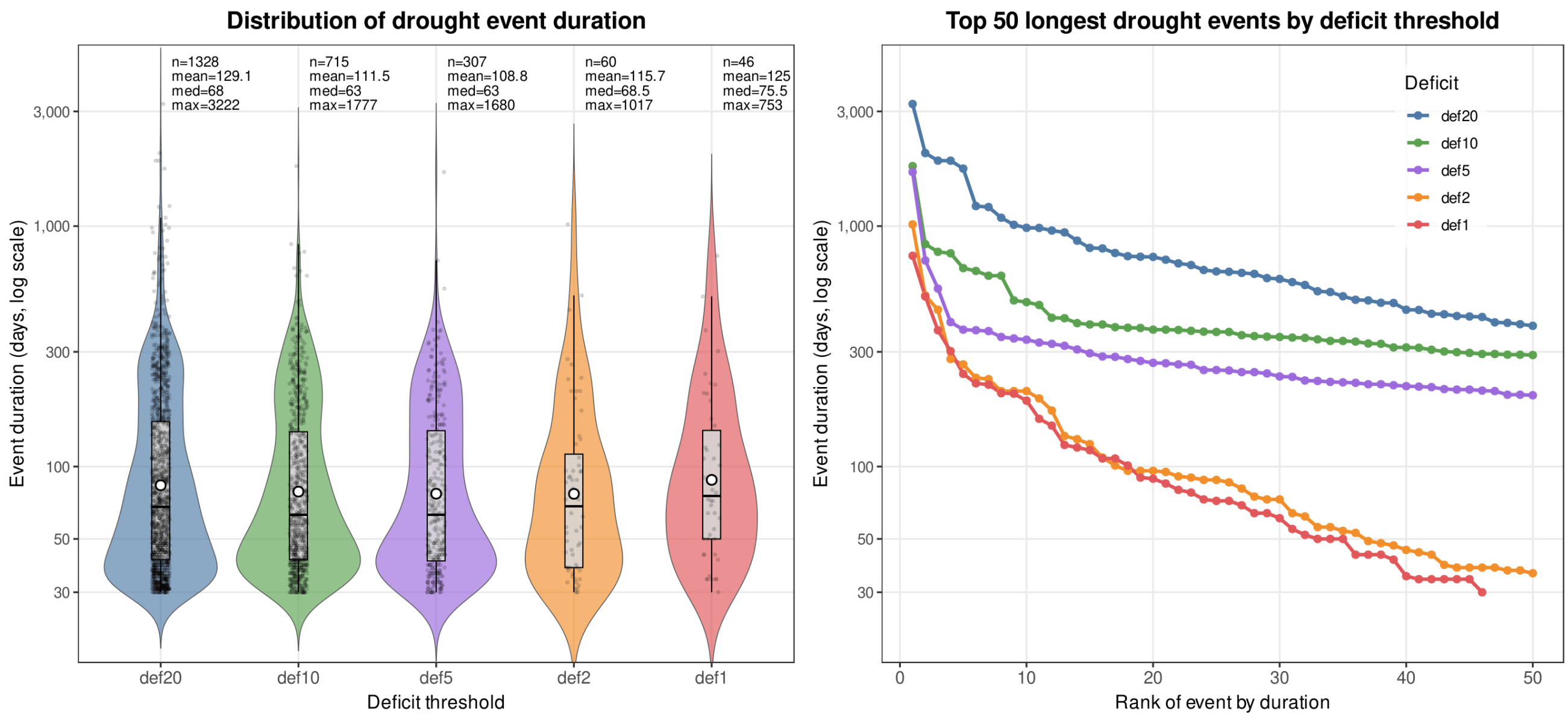

Figure S6: Duration characteristics of drought-event catalogues derived using different deficit thresholds. The left panel presents violin plots of event duration distributions, with boxplots indicating the interquartile range (25–75%), median duration, and mean duration (white circle). The right panel shows the 50 longest detected drought events ranked by duration for each deficit threshold. The figure illustrates substantial differences in event persistence and duration structure across the evaluated deficit definitions.

2.4 Cluster persistence across deficit thresholds

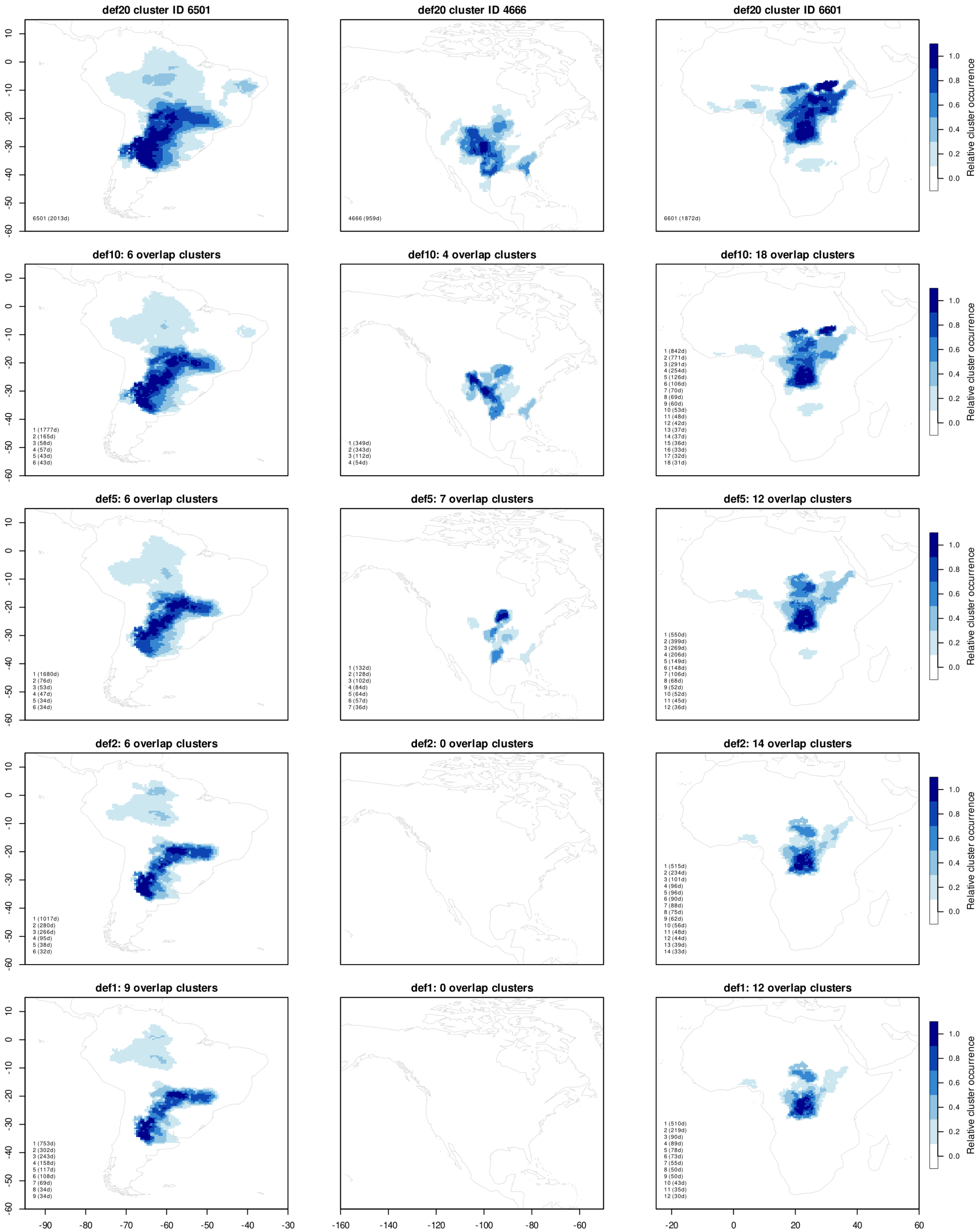

Figure S7: Comparison of representative long-duration drought clusters identified using different percentile-based deficit thresholds. Each column shows the evolution of a major drought cluster originally identified under the 20th percentile threshold (top row) and its corresponding overlapping clusters detected under stricter thresholds (10th, 5th, 2nd, and 1st percentiles). Numbers in each panel indicate the number of overlapping clusters, while values in brackets denote cluster duration in days. The figure illustrates how decreasing deficit thresholds progressively subdivide large persistent drought structures into smaller and temporally shorter events while reducing excessive temporal continuity and spatial connectivity observed under higher deficit thresholds.

Supplementary Note 3. Comparison of clustering outputs across input datasets

To evaluate the sensitivity of the clustering framework to different representations of soil moisture, we additionally generated drought event catalogues using two independent soil moisture products besides the primary mHM dataset. The first dataset was GLEAM v4.2 (Global Land Evaporation Amsterdam Model), an observationally constrained global root-zone soil moisture product available from 1980 onwards [1]. GLEAM combines satellite observations and reanalysis forcing data through a data-assimilation framework to estimate terrestrial evaporation and root-zone soil moisture. Unlike mHM, which represents soil moisture as the average water content over a fixed 2 m soil profile, GLEAM estimates root-zone moisture using a multi-layer profile whose effective depth varies according to vegetation characteristics. Daily GLEAM root-zone soil moisture data for 1980–2024 were obtained from the official GLEAM repository (`https://www.gleam.eu`).

The second dataset was SoilClim, a process-based soil-water balance model used for drought monitoring applications [2, 3, 4]. SoilClim simulates soil moisture within a fixed 2 m soil profile using meteorological forcing and soil characteristics and has previously been applied in large-scale drought assessments. For this study, we used a global SoilClim simulation covering 1980–2024. While the publicly available dataset currently extends to 2022 [5], the 2023–2024 extension was provided directly by the dataset authors to ensure consistency with the study period used throughout this catalogue.

Both GLEAM and SoilClim were originally available at 0.1° spatial resolution. To ensure direct comparability with the primary mHM-based catalogue, both datasets were aggregated to the common 0.5° grid used throughout this study using bilinear interpolation [6].

Figure S8 compares the relative frequency of drought-cluster occurrence derived from each dataset for the 1980–2024 period. The maps show the fraction of time steps during which individual grid cells were part of drought clusters lasting at least 30 days. Despite regional differences in hotspot intensity and spatial extent, the dominant large-scale drought hotspot regions remain generally consistent across datasets, particularly across North America, Europe, Siberia, southern Africa, and South America.

Table S3 summarizes the resulting catalogue characteristics for each dataset, including the total number of detected clusters, median duration, median event area, event severity, and the occurrence of persistent multi-year drought events. The mHM and GLEAM datasets produced a very similar number of drought events (516 and 506 clusters, respectively), whereas SoilClim resulted in fewer identified events (327 clusters). Despite these differences, the median event duration remained remarkably consistent across all datasets (55–59 days), and median event areas differed only moderately (349,000–415,000 km^2), indicating that the overall spatiotemporal characteristics of detected drought events are largely insensitive to the selected soil moisture dataset.

Differences were more apparent in event persistence. While mHM contained five drought events exceeding one year in duration, no such events were identified in either GLEAM or SoilClim. Conversely, GLEAM produced the highest number of short-duration events ($<$3 months), suggesting a tendency toward less persistent drought structures. These differences likely reflect variations in model structure, forcing data, and soil moisture memory among the underlying products. Nevertheless, the similarity in hotspot distributions, event durations, and spatial extents suggests that the identified large-scale drought patterns are robust across different soil moisture datasets and are not strongly dependent on the choice of a specific soil moisture product.

3.1 Sensitivity of cluster occurrence to input datasets

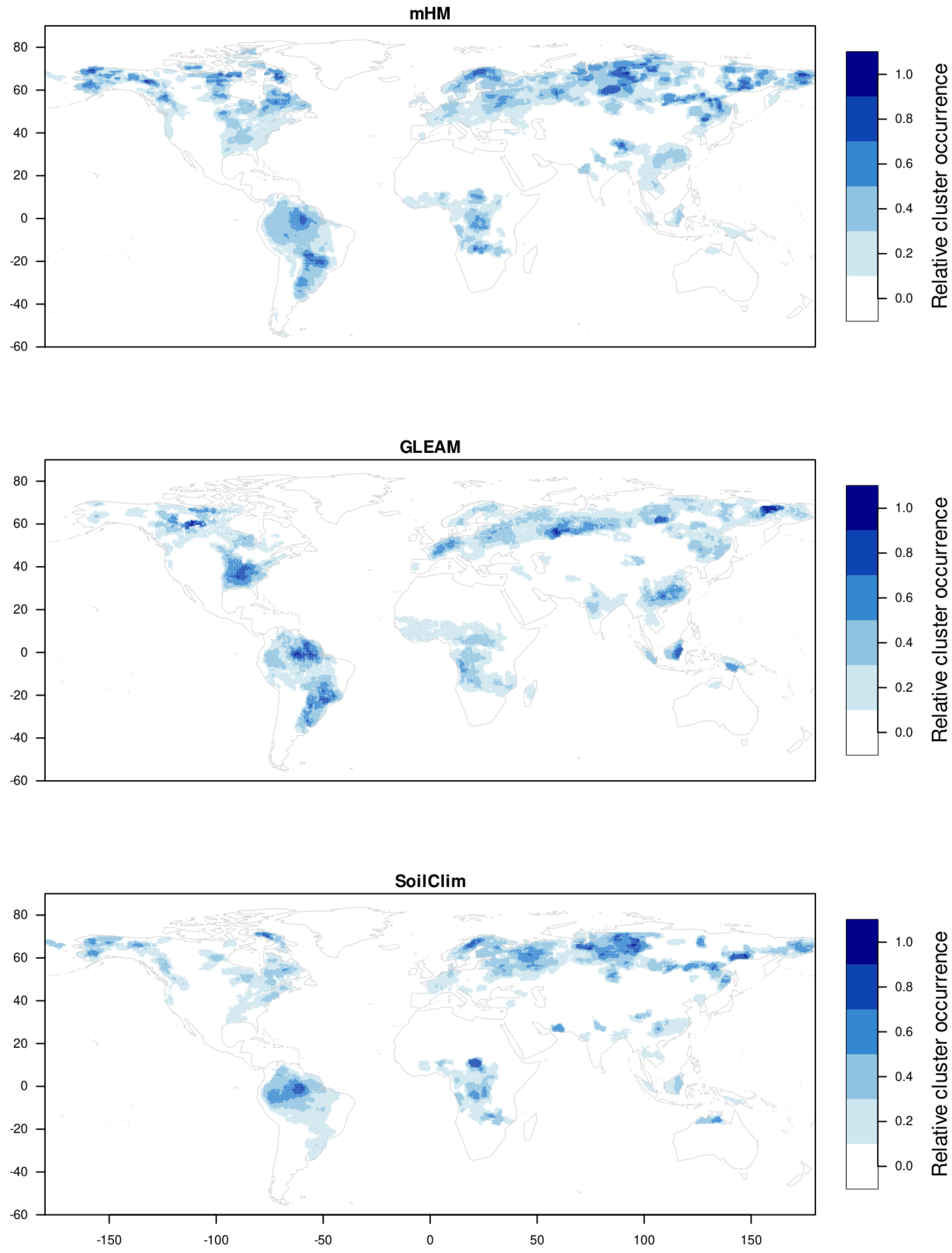

Figure S8: Relative frequency of soil-moisture drought cluster occurrence derived from three independent soil moisture datasets (mHM, GLEAM, and SoilClim) during 1980–2024. Maps show the fraction of time steps during which individual grid cells belonged to drought clusters lasting at least 30 days. All three datasets identify similar large-scale drought hotspot regions, particularly across North America, Europe, Siberia, southern Africa, and South America. The spatial patterns obtained from mHM and SoilClim are especially consistent, while GLEAM exhibits somewhat greater spatial extent and local variability in several regions. Overall, the strong agreement among datasets indicates that the major spatiotemporal drought structures identified by the clustering framework are robust to the choice of soil-moisture input data.

3.2 Summary statistics across different datasets

Table S3: Summary characteristics of drought-event catalogues derived using different soil moisture datasets. Reported metrics include the total number of detected drought events, median event duration, median maximum event area, mean event severity, and the number of persistent events exceeding one year and short-duration events shorter than three months.

Dataset	Number of clusters	Median duration (days)	Median area (km^2)	Mean severity	Events > 1 year	Events < 3 months
MHM	516	58.5	386635	39035.8	5	322
GLEAM	506	55.0	414579	23571.2	0	371
SC	327	57.0	349376	28186.8	0	232

References

[1] Miralles, D. G. *et al.* GLEAM4: global land evaporation and soil moisture dataset at 0.1 resolution from 1980 to near present. *Scientific data* **12**, 416 (2025).

[2] Hlavinka, P. *et al.* Development and evaluation of the SoilClim model for water balance and soil climate estimates. *Agricultural Water Management* **98**, 1249–1261 (2011).

[3] Trnka, M. *et al.* Czech Drought Monitor System for monitoring and forecasting of agricultural drought and drought impacts (2020).

[4] Řehoř, J. *et al.* Effects of climatic and soil data on soil drought monitoring based on different modelling schemes. *Atmosphere* **12**, 913 (2021).

[5] Řehoř, J. *et al.* Global catalog of soil moisture droughts over the past four decades. *Hydrology and Earth System Sciences* **29**, 3341–3358 (2025).

[6] Schulzweida, U. Cdo user guide (2023). URL https://doi.org/10.5281/zenodo.10020800.
